# Optimization of durum wheat bread enriched with bran

**DOI:** 10.1002/fsn3.448

**Published:** 2016-12-17

**Authors:** Maria A. Saccotelli, Amalia Conte, Krystel R. Burrafato, Sonia Calligaris, Lara Manzocco, Matteo A. Del Nobile

**Affiliations:** ^1^ Department of Agricultural Sciences, Food and EnvironmentUniversity of FoggiaFoggiaItaly; ^2^ Department of Agricultural, Food, Environmental and Animal SciencesUniversity of UdineUdineItaly

**Keywords:** bran, Durum wheat bread, organogel, textural and sensorial properties

## Abstract

An attempt is made in this study to balance the nutritional and sensory quality of bread. In particular, the formulation of a functional durum wheat bread enriched with bran at high concentration has been developed. Organogel concentration and bran particle size have been used as process variables for bread optimization. Bran concentration was increased at value as high as 15%, thus increasing the nutritional content, even though the sensory quality decreased. Bread was scored barely acceptable at 15% bran concentration. Therefore, the organogel concentration and bran particle size have been optimized to enhance bread sensory quality. Results show that it is possible to prepare bread with a significant bran enrichment without compromising its acceptability by adding proper concentration of organogel and using bran in appropriate particles size.

## 1 Introduction

Bread is a staple food generally prepared with wheat flour, water, and yeast and obtained from the total or partial baking of dough. Throughout history, this product was popular around the world and is one of the oldest food. Bread can also be prepared using many combinations and proportions of flours and other ingredients, according to different traditional recipes and preparation methods. Wheat bread accounts for about 20% of the calories consumed by humans (Brenchley, Spannagl, Pfeifer, Barker, & D'Amore, 2012).

Recently, there has been an increasing interest in wheat bread incorporating dietary fibers, such as bran (Boita et al., 2016; Boz & Karaoglu, 2013; Koletta, Irakli, Papageorgiou, & Skendi, 2014; Le Bleis, Chaunier, Chiron, Della Valle, & Saulnier, 2015). The term bran is related to the outer fibrous layer of cereal grains. It is separated from the flour through the refining process (Fulcher & Miller, 1993; Jayadeep, Singh, Sathyendra Rao, Srinivas, & Ali, 2009). Bran has attracted considerable attention due to its ability to prevent some of the most common diseases in contemporary Western society, including constipation, irritable colon, obesity, cardiovascular diseases, and colorectal cancer (Lairon et al., 2005; Schaafsma, 2004). Bran facilitates intestinal transit and stimulates colon motility; it also increases the volume and softness of stool, facilitating evacuation (Gao et al., 2009). It is useful to prevent overweight and obesity, and whenever hyper alimentation should be counteracted. Although providing a limited amount of energy, bran is characterized by a high satiety index due to its swelling capacity. It helps reducing the absorption of fat and sugar, being therefore a valuable ally to decrease the level of triglycerides and cholesterol, especially in patients with glucose intolerance or diabetes (Cavallero, Empilli, Brighenti, & Stanca, 2002; EFSA, 2010; Granfeldt, Drews, & Björck, 1995).

The incorporation of wheat bran into bread formulation necessarily modifies the properties of both dough and final bread, requiring the implementation of proper changes in processing techniques (Gan, Galliard, Ellis, Angold, & Vaughan, 1992; Lai, Davis, & Hoseney, 1989; Lai, Hoseney, & Davis, 1989; Pomeranz, Shogren, Finney, & Bechtel, 1977; Prentice & D'Appolonia, 1977). In particular, wheat bran increases dough water absorption rate, decreases dough strength, and reduces bread loaf volume (Lang, Neufeld, & Walker, 1990). These effects are the result of bran–water interactions: the excess of water absorbed in bran‐enriched dough becomes available during baking, decreasing starch gelatinization temperature and ultimately final loaf volume (Dreese & Hoseney, 1982; Lai, Hoseney, et al., 1989; Rogers & Hoseney, 1982). Hydration properties of wheat bran mainly depend on its particle size (Albers, Muchová, & Fikselová, 2009; Auffret, Ralet, Guillon, Barry, & Thibault, 1994; Zhang & Moore, 1997). Since large particles typically absorb more water than the small ones, water absorption tends to decrease by decreasing bran particle size (Auffret et al., 1994; Robertson & Eastwood, 1981; Zhang & Moore, 1997). Some studies indicated that smaller wheat bran particles provide better baking performance, while other researchers reported an opposite effect. The differences in these results could be attributed to the method of bran preparation (Brodribb & Groves, 1978; Cadden, 1986; Coda, Rizzello, Curiel, Poutanen, & Katina, 2013; Heller et al., 1980; Kirwan, Smith, McConnell, Mitchell, & Eastwood, 1974), thus suggesting that further research needs to be still carried out.

To the best of our knowledge, a very few examples of bread from durum wheat flour enriched with bran are available in the literature (Previtali et al., 2015). In any case, similar or more severe technological problems to be solved in terms of structural and sensory properties due to wheat bran addition should be also expected with durum wheat flour enriched with durum wheat bran. Therefore, proper technological solutions need to be adopted. In this context, the use of fats could represent a valid solution. In the baking process, fats are exploited to increase product volume and softness as they improve gas retention in the dough. They also act as emulsifiers and impart a desirable texture by improving heat transfer into the dough (Cauvain, 2003; Manzocco, Calligaris, Da Pieve, Marzona, & Nicoli, 2012; Shahidi, 2005). Moreover, the incorporation of lipids, shortenings and surfactants in bread dough improves bread storage quality and also influences the evolution of crumb firmness during storage (Autio & Laurikainen, 1997; Pareyt, Finnie, Putseys, & Delcour, 2011; Rogers, Zeleznak, Lai, & Hoseney, 1988; Smith & Johansson, 2004). Specifically, Rogers et al. (1988) have shown that the effects of shortenings depend on the presence of wheat flour lipids, as there is a synergistic action in terms of anti‐firming effect between shortenings and wheat flour lipids. Bread formulations generally include from 2% to 5% fat, calculated on the percentage of wheat flour (Rios, Pessanha, Almeida, Viana, & Lannes, 2014). Saturated fatty acids are usually preferred due to their intense structuring effect in bakery products. However, a high intake of saturated fatty acids is well known to cause negative health implications. Consumer demand for healthier bakery products with low fat content has thus grown significantly. In this context, an emerging strategy is based on the substitution of fat with monoglyceride gels (Batte, Wright, Rush, Idziak, & Marangoni, 2007; Krog & Larsson, 1968; Marangoni et al., 2007). When mixed with water or/and oil under given physicochemical conditions, monoglycerides can self‐assemble to beget lamellar phases (Batte et al., 2007; Heertje, Roijers, & Hendrickx, 1998; Larsson, 2009; Marangoni et al., 2007; Sagalowicz, Leser, Watzke, & Michel, 2006). The latter contribute to product structuring through the formation of a gel‐like network (i.e., organogel). The use of monoglycerides also improves dough processability, enhances initial crumb firmness and slicing performance, and reduces the staling rate (Knightly, 1988). The possibility to find their application in food products is considered one of the most interesting aspects (Da Pieve, Calligaris, Co, Nicoli, & Marangoni, 2010).

In this work, the sensory quality of durum wheat bread enriched with bran up to 15% has been optimized by acting on organogel concentration and bran particle size. In particular, the effect of both organogel and bran on bread structure and sensory quality was first assessed. Afterwards, 15% of bran enrichment in durum wheat bread was optimized with a proper concentration of organogel and selected bran particle size.

## 2 Materials and Methods

### 2.1 Materials

Myverol^™^ distilled monoglyceride (MG) (composition: total monoglyceride 97%, glycerol 0.7%, acid value 1.4) was kindly provided by Kerry (Zwijndrecht, Nederland). Durum wheat flour and bran from durum wheat of an Italian ancient cultivar (Cappelli) were used in this study for bread manufacture. Both of them were provided by the Cereal Research CREA center (Foggia, Italy). Grain was milled in a laboratory experimental mill (roller mill Mod MLU 202 Buhler; Braunschweig, Germany) to obtain durum wheat flour and bran. Compressed fresh yeast, extra‐virgin olive oil, salt, and sugar were bought from the local market.

### 2.2 Organogel preparation

Extra‐virgin olive oil organogels (OG) were prepared as described by Calligaris, Manzocco, Valoppi, and Nicoli (2013). The lipid matrix was added with 5% (w/w) monoglycerides by stirring with a magnetic road at 70°C in a water bath. The organogels were then cooled at 20°C under static conditions and used after 24 hr of storage at 20°C.

### 2.3 Bran particle size

In order to reduce the particle size of the coarse bran obtained from durum wheat milling, a further milling process was carried out. It was performed, using a Cyclotec 1093 Sample Mill (FOSS, Höganäs, Sweden), allowing the reduction of particle size, thanks to the combined action of the rotor, grinding ring, and sieve with mesh size. The particle size distribution of bran was determined by sieving 100 g of bran on a set of sieves with mesh sizes of 500, 250, 125, 63, and 25 μm. Sieving was achieved by shaking the sieves on a Retsch Vibratory Sieve Shaker AS 300 (Haan, Germany) for 30 min at an amplitude of 1.00 and subsequent delicate brushing in order to free up the clogged pores of the sieves (Jacobs, Hemdane, Dornez, Delcour, & Courtin, 2015). To calculate the particle size distribution of bran, the final mass remained on each sieve was weighted and expressed as the percentage of the total initial bran mass (100 g). In the specific, no residual mass was detected on 500‐μm sieve while on 250 μm, 125 μm and 63‐μm sieve percentages of 23.08%, 27.12%, and 49.80% were recorded, respectively. No residual mass was found on the 25 μm sieve. The bran particle size recorded on the sieve 63 μm was used for all the tests of the current work.

### 2.4 Bread‐making process

Dough mixing, processing, and baking were performed on laboratory‐scale equipment. Durum wheat dough was used as the reference sample (named as CTRL), was prepared with 1500 g of durum wheat flour, 900 g of water, 75 g of extra‐virgin olive oil, 45 g of compressed fresh yeast, 15 g of sugar, and 30 g of salt. All ingredients, except salt were mixed thoroughly with half of the water in a mixer (Conti Impastatrici, Verona, Italy) at high speed (200 rpm) for 10 min, and then the rest of the water with salt previously dissolved, was slowly added and mixed for 15 min at low speed (110 rpm). Once a homogeneous mixture was obtained, dough portions of 800 g were manually rounded and placed in a thermostatic proofing oven (Thermogel, Varese, Italy) at constant temperature (30°C) and relative humidity (85%) for about 70 min. Afterwards, the dough samples were baked in a preheated electric oven (Europa Forni, Vicenza, Italy) at 230°C for 15 min, followed by 35 min at 200°C. Samples were cooled at ambient temperature for about 2 hr before instrumental and sensory analyses. The baking process was performed in triplicate. The other enriched experimental samples were prepared using the same procedure as for the CTRL, with organogel in proper substitution of oil and bran in substitution of durum wheat flour, as shown in Table 1.

**Table 1:** Bread samples formulations

Sample	% OG	% BR	% BR‐PS
CTRL	–	–	–
OG5	5	–	–
OG5‐BR5	5	5	–
OG5‐BR10	5	10	–
OG5‐BR15	5	15	–
OG5‐BR15‐PS	5	–	15
OG7.5‐BR15	7.5	15	–
OG7.5‐BR15‐PS	7.5	–	15

All percentages were calculated on the total weight flour.

### 2.5 Textural properties

#### 2.5.1 Dough texture analysis

Texture Analyzer Zwick/Roell model Z010 (Zwick Roell Italia S.r.l., Genova, Italy) equipped with dough tensile testing device was used to measure the tensile properties. Before beginning the analysis, the material to be tested was placed between the molding and the compression plates, so that samples with suitable size were formed. After this phase, each sample was individually placed on a support plate, which was located inside the testing machine. The material testing machine starts in the tensile direction, and the tensile hook recorded the test load. Pre‐load of 0.01 N, cell load of 50 N, and a crosshead speed constant of 50 mm/min were the trial specifications (Danza et al., 2014).

#### 2.5.2 Crumb texture analysis

Compression tests were also performed using the Texture Analyzer Zwick/Roell model Z010 (Zwick Roell Italia S.r.l., Genova, Italy). Bread loaves were uniformly sliced to a thickness of 15 mm. Crust was cut off and cylindrical crumb samples (28 mm diameter) were cut with a circular cutter from the center of each bread slice. The cylindrical breadcrumb samples were placed between two parallel plates, an insert plate fixed in the universal work platform (100 × 90 × 9 mm) and compression die (75 mm diameter). For each tested sample, the force required to compress bread slices to a predetermined level against a rigid back plate was recorded, using a cylindrical plunger. A pre‐load of 0.3 N, cell load of 1 kN, maximum percentage deformation of 50%, and constant crosshead speed of 100 mm/min were the experimental conditions (Danza et al., 2014).

### 2.6 Bread sensory analysis

Bread samples were evaluated by six trained tasters. The panelists were selected on the basis of their sensory skills (ability to accurately determine and communicate the sensory attributes as appearance, odor, flavor, and texture of the product). All the instructions were given to panelists before evaluation. The panelists were also trained in sensory vocabulary and identification of attributes by evaluating durum wheat commercial bread. Specifically, the samples were compared in a crossed way with a single session panel to provide a direct comparison between various formulations and highlight the effects of individual and combined process variables. Based on these considerations, it is worth noting that each step mentioned in the R&D section, corresponds to a single panel session to allow direct comparison among samples. In particular, the panelists compared no more than two or three samples in each session, since concomitant tasting of more than three samples may cause an excessive sensory fatigue for judges, resulting in unreliable ratings.

Each sample was codified arbitrarily with three digits numbers. Before sensory analysis, samples were sliced with an electric slicing knife (thickness of 15 mm) (Atlantic; Calenzano, Firenze, Italy) without removing the crust. The bread samples were evaluated on a 9‐points scale anchored with one (extremely unpleasant), five (sensory acceptability threshold), and nine (extremely pleasant), for acceptance of seven attributes i.e., color, appearance, odor, crust and crumb firmness, presence of large bubbles, and overall quality.

### 2.7 Statistical analysis

The experimental data were subjected to statistical evaluation, using a one‐way variance analysis (ANOVA). Duncan's multiple range tests were used to determine the difference among means, and the significance was defined at *p* < .05. To this aim, a STATISTICA 7.1 for Windows (StatSoft, Inc, Tulsa, OK, USA) was used. Moreover, the interactions among textural properties of dough and crumb samples and sensory parameters of bread were evaluated, using a correlation matrix.

## 3 Results and Discussion

As reported before, an attempt is made in this work to improve the sensory quality of bran‐enriched bread by optimizing its formulation. In particular, organogel concentration and bran particle size were used as process variables to maximize the sensory quality of bread. Therefore, bran concentration was increased at value as high as 15% to increase bread nutritional value and then organogel concentration and bran particle size have been properly optimized to increase the sensory quality bread.

### 3.1 Texture analysis

#### 3.1.1 Dough mechanical properties

Table 2 shows dough mechanical properties of the investigated samples. As expected, the increase of bran concentration caused an increase of the force required to deform (*F*_*max*_) and break (*F*_*break*_) dough and a decrease in the corresponding strain (*Strain*_*max*_) and rupture (*Strain*_*break*_). The impact on dough mechanical properties may be due to physical hindrance by large bran particles, in fact, the bran can be regarded as a polymer‐based composite material which represents the reinforce phase of the dough that plays as a polymeric matrix. As the reinforce phase concentration increased, the material became more brittle. The same trend was observed when the bran particle size was reduced. The increase of the organogel concentration did not seem to significantly affect the mechanical properties of dough.

**Table 2:** Parameters of the tension test for dough samples and the compression test for crumb samples

	Dough tension test				Bread compression test
Sample	F_max_ (N)	Strain_max_ (%)	F_break_ (N)	Strain_break_ (%)	F_50%_ (N)
CTRL	0.204 ± 0.037^b,c^	23.545 ± 1.997^a^	0.102 ± 0.018^b,c^	40.625 ± 4.947^a^	5.135^ ^± 0.743^c,d^
OG5	0.166 ± 0.027^c^	22.054 ± 1.907^a^	0.083 ± 0.014^c^	40.504 ± 5.165^a^	3.875 ± 0.641^d^
OG5‐BR5	0.226 ± 0.019^b^	11.902 ± 0.892^c^	0.114 ± 0.011^b^	20.870 ± 1.611^b^	6.076 ± 0.892^b,c^
OG5‐BR10	0.204 ± 0.019^b,c^	11.613 ± 0.611^c,d^	0.102 ± 0.010^b,c^	21.707 ± 2.468^b^	5.931 ± 0.968^b,c,d^
OG5‐BR15	0.299 ± 0.019^a^	11.081 ± 0.641^c,d^	0.149 ± 0.010^a^	17.513 ± 1.537^b,c^	7.727 ± 1.514^b^
OG5‐BR15‐PS	0.238 ± 0.017^b^	11.532 ± 0.553^c,d^	0.118 ± 0.008^b^	20.518 ± 1.790^b,c^	10.781 ± 1.895^a^
OG7.5‐BR15	0.303 ± 0.027^a^	9.433 ± 0.626^d^	0.152 ± 0.013^a^	15.132 ± 1.695^c^	7.818 ± 0.888^b^
OG7.5‐BR15‐PS	0.230 ± 0.032^b^	14.295 ± 1.051^b^	0.115 ± 0.016^b^	22.849 ± 2.356^b^	5.308 ± 0.950^c,d^

^a‐d^Means in the same column followed by different superscript letters differ significantly (*p* < .05).

#### 3.1.2 Crumb mechanical properties

The *F*_*50%*_ data for the investigated samples are also listed in Table 2. As can be inferred from data listed in the above table, there was a substantial positive effect of either oil (CTRL) or organogel (OG5) on crumb softness. As expected, the addition of bran promoted an increase in crumb firmness. Data also show that increasing bran concentration, in the range investigated in this work, did not significantly change crumb softness. Increasing organogel concentration seemed to affect the mechanical properties of the crumbs only if small bran particles were used. In this case, a decrease in *F*_*50%*_ was observed as the organogel concentration increased. The effect of bran particle size results was not statistically significant at high organogel concentration, whereas at low organogel concentration an increase in *F*_*50%*_ was observed, as the bran particle size was reduced.

### 3.2 Bread sensory properties

In Table 3, the sensory characterization of bread added with oil, organogel, and bran is reported. The substitution of oil by organogel made in Step 1 increased the mean value of bread sensory quality, in terms of softness and porosity. By contrast, the addition of bran to the formulation containing organogel decreased the mean values of crumb softness and porous size to values lower than those of the control bread prepared with oil. Monoglycerides are examples of crumb softeners that may improve dough and increase tolerance to mixing (Calligaris et al., 2013; Manzocco et al., 2012; Pareyt et al., 2011). They can also improve the retention of gas and the final loaf volume. For this reason, they are preferred in bread formulations to replace part of the fat and improve lipid dispersion (Pareyt et al., 2011). On the contrary, the addition of bran reduces the sensory quality of bread as it interferes with the network formation. The incorporation of bran in foods generally results in inferior end product quality (Hemdane et al., 2015). In another study, it was shown that addition of wheat bran up to 5% resulted in a decrease of bread volume, arising from dilution of gluten proteins (Pomeranz et al., 1977), further confirming that bread sensory quality is negatively affected by bran addition. In fact, wheat bran alters the network formation and destabilizes the gas cells causing low gas retention (Gan et al., 1992; Pomeranz et al., 1977).

**Table 3:** Sensory characteristics of bread samples

Sample	Color	Appearance	Odor	Crust firmness	Crumb firmness	Large bubbles	Overall quality
Step 1							
CTRL	7.25^ ^± 0.27^a^	7.33^ ^± 0.41^a^	7.25^ ^± 0.42^a^	7.17 ± 0.26^a^	7.25 ± 0.52^a^	7.08 ± 0.20^b^	7.25 ± 0.27^a,b^
OG5	7.33 ± 0.26^a^	7.42 ± 0.20^a^	7.33 ± 0.52^a^	7.25 ± 0.27^a^	7.83 ± 0.41^a^	7.75 ± 0.27^a^	7.83 ± 0.26^a^
OG5‐BR5	7.08 ± 0.38^a^	7.17 ± 0.26^a^	7.00 ± 0.45^a^	7.08 ± 0.49^a^	7.17 ± 0.52^a^	6.67 ± 0.41^b^	7.17 ± 0.41^b^
Step 2							
OG5‐BR5	7.25 ± 0.27^a^	7.08 ± 0.38^a^	7.25 ± 0.42^a^	7.17 ± 0.41^a^	7.00 ± 0.00^a^	6.67 ± 0.26^a^	7.17 ± 0.26^a^
OG5‐BR10	7.17 ± 0.41^a^	7.00 ± 0.45^a^	7.25 ± 0.27^a^	7.08 ± 0.20^a,b^	6.25 ± 0.27^b^	6.00 ± 0.00^b^	6.50 ± 0.32^b^
OG5‐BR15	6.50 ± 0.45^a^	6.42 ± 0.20^a^	7.17 ± 0.26^a^	6.50 ± 0.32^b^	5.25 ± 0.42^c^	5.17 ± 0.26^c^	5.25 ± 0.27^c^
Step 3							
OG5‐BR15	6.50 ± 0.00^b^	6.33 ± 0.41^b^	7.08 ± 0.20^b^	6.33 ± 0.26^b^	5.00 ± 0.32^a^	5.17 ± 0.41^a^	5.08 ± 0.20^b^
OG5‐BR15‐PS	7.33 ± 0.26^a^	7.25 ± 0.27^a^	7.75 ± 0.27^a^	7.25 ± 0.42^a^	5.58 ± 0.38^a^	5.50 ± 0.32^a^	6.20 ± 0.27^a^
Step 4							
OG5‐BR15	6.58 ± 0.20^a^	6.42 ± 0.49^a^	7.25 ± 0.42^a^	6.42 ± 0.20^a^	5.25 ± 0.27^b^	5.08 ± 0.38^b^	5.17 ± 0.26^b^
OG7.5‐BR15	7.00 ± 0.32^a^	7.08 ± 0.38^a^	7.83 ± 0.26^a^	6.50 ± 0.45^a^	6.25 ± 0.42^a^	6.17 ± 0.26^a^	6.50 ± 0.32^a^
Step 5							
OG7.5‐BR15	7.17 ± 0.41^b^	7.08 ± 0.20^b^	7.67 ± 0.41^a^	6.33 ± 0.26^b^	5.75 ± 0.27^b^	5.42 ± 0.20^b^	6.25 ± 0.42^b^
OG7.5‐BR15‐PS	8.00 ± 0.00^a^	8.00 ± 0.00^a^	8.00 ± 0.32^a^	7.75 ± 0.27^a^	7.17 ± 0.26^a^	6.67 ± 0.26^a^	7.42 ± 0.20^a^

For each Step: ^a‐c^ Means in the same column followed by different superscript letters differ significantly (*p* < .05).

Bread samples were compared as follows: Step 1: control bread (CTRL), bread containing 5% organogel (OG5) and bread containing 5% organogel with 5% bran (OG5‐BR5); Step 2: bread containing 5% organogel and 5%, 10% or 15% bran (OG5‐BR5, OG5‐BR10, OG5‐BR15); Step 3: bread containing 5% organogel and 15% bran (OG5‐BR15) or 15% bran at reduced particle size (OG5‐BR15‐PS); Step 4: bread containing 5 or 7.5% organogel and 15% bran (OG5‐BR15, OG7.5‐BR15); Step 5: bread containing 7.5% organogel and 15% bran (OG7.5‐BR15) or 15% bran at reduced particle size (OG7.5‐BR15‐PS).

Step 2 of Table 3 shows the results for samples containing 5% organogel and increasing concentrations of bran up to 15%. As expected, data of panel test confirm that the increase in bran concentration decreased the mean value of color, appearance, and odor. Crumb firmness and porous structure were significantly impaired by bran addition so that the bread containing 15% bran was scored barely acceptable from the overall sensory point of view. To improve the sensory quality of bread containing 15% bran, first proper bran particle size (63 μm) was selected (Step 3) and then the organogel concentration was increased from 5 to 7.5% (Step 4). As reported in the Table, data of Step 3 and 4 highlight that both particle size and organogel improved bread sensory acceptability, respectively. In the final step (Step 5), the maximum organogel concentration and the selected bran particle size were combined and properly compared. From the Table, it can be observed that both the decrease in bran particle size and the increase in organogel concentration were associated to an increase in the score of all bread attributes, resulting in a bread with highly potential nutritional properties and very comparable to the control sample from the sensory point of view.

### 3.3 Correlations

Table 4 shows the correlations among the main sensory attributes and the main mechanical properties of both crumb and dough. Crumb firmness, large bubbles, and overall quality assessed by sensory analysis resulted strictly correlated with the instrumental parameters *Strain*_*max*_ and *F*_*50%*_. In particular, increasing the crumb mean porous size increases softness as well. These last two sensory attributes (i.e., crumb softness and crumb pore size) are also positively correlated with the overall quality of bread, highlighting that crumb structure plays a major role in determining the bread overall quality. Data listed in Table 4 also show that dough elasticity (*Strain*_*max*_) was positively correlated with the sensory attributes of bread. This is because an elastic dough allows bubbles to grow and form an aerated structure. As one would expect, the crumb mechanical property (*F*_*50%*_ *)* was negatively correlated with crumb softness and crumb porous size, and consequently to overall quality of bread.

**Table 4:** The correlations among sensory and textural parameters of bread samples

	Crumb firmness	Large bubbles	Overall quality	Strain_max_	F_50%_
Crumb firmness	1	0.9799	0.9719	0.7109	−0.8359
Large bubbles	__	1	0.9493	0.7861	−0.8172
Overall quality	__	__	1	0.6576	−0.7211
Strain_max_	__	__	__	1	−0.6565
F_50%_	__	__	__	__	1

Parameters evaluated were highly and significantly correlated.

## 4 Conclusion

This work addressed the improvement of the sensory quality of bran‐enriched durum wheat bread by acting on formulation. In particular, bran was added to a concentration as high as 15% to raise the nutritional quality of bread, whereas organogel concentration and bran particle size were used as process variables to improve bread sensory quality. Results show that bran concentration, while improving the bread nutritional value, strongly reduces its sensory quality; in fact, the score of bread at 15% bran concentration was near to sensory acceptability threshold. On the other hand, the addition of organogel and the decrease of bran particle size positively affect crumb porous size and softness, improving bread sensory quality. It is worth noting that the same approach used in this work can find application in the production of other bakery products such as fiber‐enriched cakes and biscuits, in which fat could be replaced by various forms of lipid structures (e.g. monoglycerides self‐assembly structures, organogels) to improve the rheological and sensory properties of both the dough and the final product.

## Conflict of Interest

None declared.
